# Direct and Indirect Effects of Blood Levels of Omega-3 and Omega-6 Fatty Acids on Reading and Writing (Dis)Abilities

**DOI:** 10.3390/brainsci12020169

**Published:** 2022-01-27

**Authors:** Francesca Borasio, Marie-Louise Syren, Stefano Turolo, Carlo Agostoni, Massimo Molteni, Alessandro Antonietti, Maria Luisa Lorusso

**Affiliations:** 1 Unit of Child Psychopathology, Scientific Institute IRCSS E. Medea, 23842 Bosisio Parini, Italy; francesca.borasio@lanostrafamiglia.it (F.B.); massimo.molteni@lanostrafamiglia.it (M.M.); 2 Department of Psychology, Catholic University of the Sacred Heart, 20123 Milano, Italy; alessandro.antonietti@unicatt.it; 3 Department of Clinical Sciences and Community Health, University of Milan, 20123 Milano, Italy; eva.syren@unimi.it (M.-L.S.); carlo.agostoni@unimi.it (C.A.); 4 UOC Nephrology Dialysis and Pediatric Transplantation, Fondazione IRCCS Ca’ Granda Ospedale Maggiore Policlinico, 20123 Milano, Italy; stefano.turolo@policlinico.mi.it; 5 Pediatric Unit, Fondazione IRCCS Ca’ Granda Ospedale Maggiore Policlinico, 20123 Milano, Italy

**Keywords:** PUFA, dyslexia, reading, writing, visual processing, phonological processing, visual-spatial attention, cueing task, flanker effect

## Abstract

The purpose of the present study was to investigate whether there are associations between polyunsaturated fatty acid (PUFA) blood levels, reading/writing performance and performance in neuropsychological tasks. Moderate to strong correlations were found between PUFA levels (specific omega-6/omega-3 ratios) and reading/writing abilities, and the former and neuropsychological test scores. Mediation models analyzing the direct and indirect effects of PUFA on reading and writing scores showed that the effects of fatty acids on learning measures appear to be direct rather than mediated by the investigated visual and auditory neuropsychological mechanisms. The only significant indirect effect was found for the difference in accuracy between the left and right visual fields in visual-spatial cueing tasks, acting as a mediator for the effect of PUFA ratios on writing accuracy. Regression analyses, by contrast, confirmed the roles of phonological awareness and other visual attentional factors as predictors of reading and writing skills. Such results confirm the crucial role of visual-spatial attention mechanisms in reading and writing, and suggest that visual low-level mechanisms may be more sensitive to the effects of favorable conditions related to the presence of higher omega-3 blood levels.

## 1. Introduction

Developmental dyslexia (DD) is one of the most common learning disabilities. It is characterized by deficiencies in the acquisition of reading skills, despite there being no evidence of intellectual disability, or sensory or neurological deficiencies [1,2]. DD is a heterogeneous disorder, and many researchers—e.g., [3,4]—agree with the idea that several factors contribute to explain the emergence of reading disorders.

Over the last few decades, indeed, deficits in reading were shown to be associated with phonological processing [5,6] and perceptual or visuo-attentional mechanisms [7,8,9,10]. Some children with DD are unable to focus their visual attention normally or to elaborate the spatial relationships of visual information in a specific region of space [11,12]. These processes are often linked to the magnocellular system, i.e., the part of the visual system specialized in the processing of low-spatial-frequency and high-temporal-frequency stimuli.

The magnocellular system (also known as the dorsal system or dorsal visual stream) can be described as a circuit, including different brain regions, which is dedicated to space perception, but also to the coding of visual information for action organization [13]. Since the system projects also to the PPC (posterior parietal cortex) and to the cerebellum, it is involved in the control of multisensory selective attention, eye movements and motion processing [9,14,15,16]. More specifically, the attentional systems that form part of the magnocellular system, and have been shown to be also involved in reading, control the ability to concentrate visual attention in a restricted area of the visual field and to shift it when needed, but also the so-called visual crowding, a mechanism of the perceptual system producing a sort of “blurring” of the visual areas surrounding the target object to be analyzed, resulting in the masking of further visual elements that may be present in those areas [17,18].

Several children with DD display reduced motion sensitivity and impaired focusing of visual or auditory attention [11,19,20]. The basic mechanisms of visual attention can also be considered to play a role in early stages of grapheme-to-phoneme conversion, and they were shown to be involved in visual search and in graphemic parsing through neuronal-oscillation modulation mechanisms very similar to those involved in phonological processing according to the Temporal Sampling Framework (TSF) proposed by Goswami [19,21]. In other words, the TSF could be applied to the various stages of processing within the visual system as well, prior to the information entering the phonological processing stage [19].

Omega-6 and omega-3 polyunsaturated fatty acids (PUFA) play an important role in physiological brain development and function [22,23]. PUFA are involved in membrane fluidity, gene expression, and neuronal membrane structure and function, and are critical elements for cell transduction and learning processes [24]. Some studies suggested that there is a link between defects in the metabolism of PUFA and neurodevelopmental disorders, and that some children with DD are deficient in PUFA [25,26,27].

Long-chain omega-3 and omega-6 PUFA are synthesized from the essential fatty acids (EFA) α-linolenic acid (ALA, 18:3ω-3) and linoleic acid (LA, 18:2ω-6), respectively [28]. These acids are synthesized in plants, so it is possible to find them in high proportions in plant-based foods [29]. Animals and humans have the capacity to metabolize EFA to long-chain derivatives. As the omega-6 and omega-3 pathways compete with one another for enzyme activity, the ratio of omega-6 to omega-3 PUFA is very important to human health [30]. An overabundance of fatty acids from one family will limit the metabolic production of the longer chain products of the other. The typical Western diet is characterized by higher intake of LA compared to ALA (by 5 to 15 times higher) [31], and it provides omega-6 and omega-3 PUFA in a ratio ranging from 8:1 to 25:1, values in contrast to the recommendations of approximately 4:1 from the national health agencies [30]. Lowering the omega-6 to omega-3 ratio would reduce competition for the enzymes and facilitate the metabolism of more downstream products of ALA [30].

Omega-3 PUFA, in particular, have important roles in the brain beyond infancy [32] and possess antioxidant and anti-inflammatory properties [33,34]. The omega-6 PUFA LA and AA (arachidonic acid, 20:4ω-6), instead, are mainly pro-inflammatory in character. However, there seems to be a limited effect on inflammatory biomarkers at the intake levels currently consumed. Furthermore, AA is important in brain development and cognitive function [29]. It is widely accepted that an optimal ratio of omega-6 to omega-3 PUFA positively affects biological processes such as inflammation, although there is no consensus on what that exactly ratio should be. Moreover, the relationship between omega-6 and omega-3 PUFA and their role in the context of inflammation are not yet clear [29]. The omega-6 to omega-3 ratio has been found to play an important role in cardiovascular health and in other chronic illnesses [35], but it has been shown to play a role also in neurodevelopmental disorders such as ADHD and autism spectrum disorders [36,37], and in brain development [38], where it turned out to be negatively associated with performance on executive functions and planning skills in 7 to 9 year-old children, possibly mediated by an enzymatic affinity for n-3 fatty acids.

Several studies investigated the levels of blood fatty acids in children with DD, and it was found that children with the most severe fatty acid deficiencies had poorer reading, spelling and auditory working memory than children with milder PUFA deficiency [39]. Moreover, omega-3 appears to be associated with reading and spelling ability, regardless of the diagnosis of DD [25]. Long chain PUFA have been shown to improve information processing speed [40], learning and visual memory [41,42], and visual attention [43,44,45]. However, some studies failed to find any robust effects of PUFA on cognition [46] and the great variation in study design, type of PUFA, participant characteristics, and measuring techniques make it difficult to draw any clear conclusion from the literature.

The aim of the present study was to investigate the correlations between PUFA blood levels, reading/writing performance and performance in neuropsychological tasks. More precisely, our aim was to test the hypothesis that the effect of PUFA on reading and writing skills is mediated by neuropsychological functions such as phonemic awareness, magnocellular-related visual perception, visual-spatial attention (VSA), and executive functions.

Based on previous studies on PUFA and learning and neuropsychological abilities, our first hypothesis was that high levels of omega-6 and low levels of omega-3 and/or low omega-6 to omega-3 ratios would be associated with worse performance in reading and writing. Secondly, we expected that such associations could be explained (mediated) by the effects of PUFA levels on the neuropsychological functions for which speed of processing is more crucial, i.e., low-level perceptual functions (such as perception of rapid movement in the visual modality and discrimination of rhythmic patterns or of phoneme-level differences in the auditory modality). Our third hypothesis was that the influence of visual and auditory perception could be exerted at high levels of processing—not just basic perceptual tasks (such as perception of rapid movement in the visual modality and discrimination of rhythmic patterns or of phoneme-level differences in the auditory modality), but more complex tasks involving visual searching (for the visual modality) and phonological awareness (for the auditory modality). Therefore, visual search tasks and phonological awareness were included in the neuropsychological battery to allow investigation of their possible roles as mediators. Indeed, phonological awareness is known to play an important role in reading and DD: some children with reading disabilities have difficulties in segmenting the phonemic constituents of words, or in matching speech sounds with the visual counterparts [47,48,49]. Since PUFA supplementation had been found to improve both phonological and visual processing in typically developing children [50], our fourth hypothesis concerned the possible involvement and mediating role of transmodal abilities related to the association of verbal labels to visually presented stimuli, as assessed by RAN (rapid auditory naming), another function that has been shown to be closely associated with reading and DD [47,51].

The study is situated in the context of a larger trial on the effects of PUFA supplementation during intervention for DD, which included assessments with further tasks that will not be considered in the present study.

## 2. Materials and Methods

### 2.1. Participants

A total of 30 Italian students aged between 8 and 13 years (mean age = 10.83 years, SD = 1.43) were involved in the study. The sample consisted of 15 children diagnosed with DD and 15 normally reading children. The recruitment took place between July 2020 and September 2021. Participants with reading disorders were selected among patients of the neuropsychiatry unit of IRCCS “Eugenio Medea” in Bosisio Parini, Northern Italy, and typically developing children (TD) were recruited in the same geographic area among the schoolmates and friends of the children with DD, through sport centers or through social media. The children and their families were contacted by the researchers, and the purpose and procedures of the study were explained. Written parental informed consent was obtained before the beginning of the study.

Participants had to fulfil the following inclusion criteria: (a) between 7 and 15 years of age and attending at least the third class of primary school; (b) IQ ≥ 80; (c) monolingual speakers or bilingual speakers with perfect (native-like) mastery of the Italian language. Moreover, inclusion criteria for children with DD were: (a) having been previously diagnosed with DD on the basis of standard inclusion/exclusion criteria (ICD-10: World Health Organization, 1992); (b) absence of comorbidity with ADHD and other neuropsychiatric or psychopathological conditions (whereas comorbidity with other learning disorders was allowed); (c) not having received neuropsychological treatment for DD before. Inclusion criteria for TD children were: (a) normal school achievement as reported by teachers and parents; (b) no z-scores below −1.5 with respect to age mean in text, word, and nonword reading tests and in tests of writing to dictation (DDE-2 battery, MT reading test). The study was approved by the Local Ethics Committee in accordance with the Declaration of Helsinki.

### 2.2. Measures and Procedure

The study is part of an ongoing project on the efficacy of PUFA supplementation to enhance the effects of neuropsychological treatment for children diagnosed with DD (registered in ClinicalTrials.gov, Code NCT04287530).

All measures involved in the present study were collected as part of the pre-test assessment of the main project. Reading and neuropsychological tests were administered individually by trained psychologists in one session of about an hour and a half. The blood samples were collected by nurses of the Scientific Institute Eugenio Medea during the same assessment session.

#### 2.2.1. Blood Measurements of Fatty Acids

The methodology described in Marangoni et al. [52] was used to analyze the blood levels of PUFA. Blood samples were obtained by collecting a drop of blood from a fingertip. Samples were collected on Whatman 903 collection cards BHT pre-treated and stored at a temperature of −20 °C. The dried blood spot was methylated with HCl/MeOH (Supelco, MERCK), the fatty acid methyl esters (FAME) were extracted with hexane and injected into Shimadzu Nexis GC-2030 gas cromatograph. A 30 m capillary column (FAMEWAX, RESTEK) was used to separate the FAME. The Labsolution software (Shimadzu) was used to identify FA species using the retention time of standards (PUFA1, PUFA2, PUFA3 (Supelco, MERCK) and NHI-F (AccuStandard, RESTEK).

Total PUFA, total omega-3, total omega-6 compositions were measured as a percentage of the total fatty acid. The ratios omega-6/omega-3, AA/EPA (eicosapentaenoic acid, 20:5ω-3), AA/DHA (docosahexaenoic acid, 22:6ω-3), AA/ALA, LA/ALA were also computed.

#### 2.2.2. Neuropsychological Tests

All participants were tested before starting treatment. Assessment involved reading skills and a set of neuropsychological functions related to the process of reading. The results of all the tests are expressed as both raw scores and z-scores according to age norms, with the exception of the motion coherence test, the rhythmic pattern discrimination, and the cue effects in visual spatial attention, for which norms are not available, and therefore, only raw scores could be recorded. The following tests were administered:

(a) *Cognitive measures*. The Wechsler Intelligence Scale for Children, Fourth Edition (WISC-IV) [53] and the Raven’s Coloured Progressive Matrices Test (CPM) [54,55] were adopted to assess inclusion criteria related to normal intelligence. verbal comprehension, perceptual reasoning, working memory, and processing speed indexes were calculated in addition to full scale IQ (FS-IQ) for the WISC-IV. The WISC-IV was part of the clinical assessment for children diagnosed with DD, whereas the CPM test was used for TDchildren.
(b) *Reading and writing tests*.

(b1) Single word/nonword reading. “DDE-2: Batteria per la Valutazione della Dislessia e Disortografia Evolutiva-2” (Assessment battery for Developmental Reading and Spelling Disorders-2) [56] was used to assess speed and accuracy (expressed in number of errors) in reading word and nonword lists. It provides grade norms from the second to the last grade of junior high school.

(b2) Word and sentence writing to dictation. Two dictation tasks were taken from the DDE-2 battery [56], giving accuracy scores (number of errors) according to age norms in writing (48) words, and (12) sentences.

(c) *Neuropsychological tests: auditory*.

(c1) Rhythmic pattern discrimination. The pattern discrimination task proposed by Cantiani and colleagues [57] was adopted to assess rhythmic pattern discrimination. Children were required to discriminate four-tone rhythmic patterns. The stimulus patterns consisted of four tones separated by different ISIs (50 ms; 150 ms; 200 ms). The synthetically generated tones were 500 Hz in frequency and 30 msec in duration. Two different stimulus patterns (rhythms) were created, by changing the order of the ISIs, and the two rhythms were paired in all four possible combinations (AA, AB, BB, BA) with 700-ms intervals. The children listened to the pairs of rhythms and were instructed to indicate, by an oral answer, if the two rhythms were equal or different. The number of correct answers (Pattern total), and responses to different rhythms (Pattern different) and responses to equal rhythms (Pattern equal) were analyzed separately.

(c2) Phonological awareness. A subtest of the Developmental Neuropsychological Assessment, Second Edition (NEPSY-II) [58] was used to assess phonological processing skills. This test is subdivided into two parts. In part A children are required to establish a correspondence between sounds and images, and to select the corresponding image based on a partial sound. Part B requires the child to encode a verbal stimulus and to manipulate its phonetic structure by either removing a sound, or by replacing a sound with another one. Raw scores are calculated as the number of correct answers (range 0–53), and z-scores are computed according to the age norms.

(c3) Rapid automatized naming. “Denominazione Rapida di Colori” (rapid automatized naming test—RAN for Colors) [59] was used to assess the naming speed for familiar stimuli (colors). Two matrices (10 rows of 5 stimuli each) of colored squares (i.e., black, blue, red, yellow, and green) were presented and the child was asked to sequentially name each visual stimulus of the matrix as quickly and as accurately as possible. Two raw scores were recorded: speed (expressed in seconds) and accuracy (expressed in number of naming errors). Z-scores based on the grade norms were calculated.

(d) *Neuropsychological tests: visual*.

The following two tests of VSA were administered.

(d1) The flanker (FL) task was administered to assess cognitive inhibition. This test is taken from the Amsterdam Neuropsychological Tasks (ANT) [60], a set of computerized tests. Participants were seated approximately 50 cm in front of the computer screen and responses were given by pressing the left or right mouse button with their dominant hand. In this task a central target stimulus, surrounded by eight distractors (flankers), is presented in the middle of the screen. The color of the target stimulus is linked to the answer button: If it is blue, then the left button must be pressed; If it is yellow, then the right button should be pressed. The flanker task is made of two parts. Part 1 consists of 40 trials with compatible flankers (same color as the central stimulus) and neutral flankers (color is different from the color of the central stimulus). Part 2 consists of 80 trials with the target stimulus surrounded by compatible or incompatible flankers (color that was associated with the opposite response). In Part 2, children were required to answer exactly as required in Part 1, pressing the left button if the target stimulus is blue, or pressing the right one if the target stimulus is yellow. The flanker effect was calculated by subtracting the mean reaction time on compatible trials from the mean reaction time on incompatible trials.

(d2) Cue effects in VSA. A classical Posner’s cueing paradigm for measuring covert visual spatial attention (VSA) was constructed following exactly the same procedure described in Facoetti et al. [12]. The fixation point consisted of a cross (18 of visual angle) appearing at the center of the computer screen, and two circles (2.5°) were presented peripherally (8° of eccentricity), one to the left and one to the right of the fixation point. The target (a dot of 0.58°) was preceded by a spatial cue (1.58° arrow appearing in the center or in the periphery), which could be valid (80% of the trials) or invalid (20% of the trials). Children were instructed to keep their eyes on the fixation point throughout the duration of the experimental session. Each trial started with the onset of the fixation point accompanied by a 1000-Hz warning signal tone. After 500 ms the two circles were displayed peripherally and 500 ms later the cue was shown for 50 ms. After 300 ms the target appeared for 50 ms inside one of the two circles: On valid trials, the target was presented inside the circle pointed by the arrow cue, whereas on invalid trials the target appeared in the circle on the opposite side. Children were instructed to react as quickly as possible by pressing the spacebar on the computer keyboard. The maximum time allowed for responding was 1500 ms and the intertrial interval was 1000 ms. Catch trials, in which the target was not presented, were intermingled with response trials. The experimental session consisted of 192 trials divided into two blocks (one of central cues and one of peripheral cues) of 96 trials each. Block sequence was counterbalanced within participants and trials were distributed as follows: 64 valid trials (32 on each side), 16 invalid trials (8 on each side), and 16 catch trials. The cue effect was calculated by subtracting the mean reaction time on valid condition trials from the mean reaction time of invalid conditions trials. The left–right difference in VSA cue effect was computed by subtracting the cue effect in the right visual field from the cue effect in the left visual field. Left–right differences in accuracy and speed for the VSA cue effect were calculated.

(d3) Motion coherence. Motion perception was evaluated with the Motion Coherence test presented in Benassi et al. [61]. The stimulus consisted of 150 high-luminance dots (luminance = 51.0 cd/m^2^, dot diameter = 3 arcmin, dot density = 1.25 dots/deg^2^) that could either move coherently at a constant speed (1.5 deg/s) in one of eight directions in the space (four cardinal and four oblique) or in a Brownian manner (noise dots) within a circular frame of 6.2 deg on a black background (0.2 cd/m2). The present test consisted of high-temporal-frequency stimuli that moved across the screen and required children to identify the direction in which the coherent dots were moving. The stimulus was presented on the screen for 1000 ms; Then it disappeared and all eight possible directions appeared on the screen, indicated by eight grey arrows. Children were required to indicate the direction of the coherent moving dots by clicking with the mouse on the indicated arrow. The test consisted of five levels, corresponding to five levels of coherence, and each level was composed of eight trials. The first level started from a condition of 100% coherence (all dots moved coherently in one specific direction; no noise), and at each level the coherence rate decreased by 2 dB (100%, 63.10%, 39.81%, 25.12%, and 15.85%). The global score was calculated as the number of correct responses (range 0–44).

(d4) Visual search. “Ricerca visiva di Colori” (visual search—Colors) [59] was used to assess speed and accuracy in visual search for familiar stimuli (colors). Two matrices (10 rows of 5 stimuli each) of digit (i.e., 2, 4, 6, 7, and 9) were presented and the child was requested to cancel one of the stimuli (the number 7) presented in the matrix as quickly and as accurately as possible. Two raw scores were recorded: speed (expressed in seconds) and accuracy (expressed in number of cancellation errors). Z-scores based on the grade norms were calculated.

### 2.3. Data Analysis

Preliminarily, reading and writing performances (raw scores) were analyzed in children with and without DD. A series of independent samples *t*-tests were performed in order to ensure that the children of the two groups differed in all and only the measures related to diagnostic criteria. Correlational analyses were performed considering the entire sample of children (n = 30). This choice was met to maximize statistical power, but also following the observation that reading and writing scores in the sample were rather homogeneously distributed along a continuum (see Figure S1) and could thus well represent an extended window on reading and writing skills viewed as general, complex multifactorial skills resulting from the interplay of several factors [3,4,62] and not from the expression of a single process.

Power analysis (G*Power [63]) was conducted with respect to multiple linear regression coefficients using three predictors’ models: Based on previous data and allowing an effect size of 0.32 [25] for the expected correlation data for LA/ALA with reading and spelling scores, 30 participants were required to reach a power of 0.80.

First of all, the associations of raw scores from reading and writing tests and the levels or ratios of PUFA in the blood were analyzed through partial correlation analyses with age as a control variable. Based on emerging correlations, the associations of selected reading and writing measures with visual/auditory neuropsychological tasks were analyzed. In the last step of the correlation analysis, the associations between the auditory and visual neuropsychological functions identified in the previous step with PUFA measures that had been found to be related to reading and writing were finally analyzed.

Standard GLM Mediation models (Jamovi [64]) were adopted to test the possible mediating role of neuropsychological functions (those found to be significantly associated with reading/writing performance) in the relationship between PUFA and reading/writing skills. Z-scores were used for all measures in the mediation models, with the exception of PUFA ratios and VSA cue effects. Since PUFA and VSA cue effects did not correlate significantly with age (all *p* > 0.232), and based on previously observed partial correlation patterns, age was not entered in the models.

For all the analyses, three general scores were computed for reading and writing measures based on raw scores: (1) DDE general reading time score, i.e., the average of time used for word and nonword reading (expressed in seconds), (2) DDE general reading errors score, i.e., the average number of errors in word and nonword reading, and (3) DDE general writing errors, i.e., the average number of errors in word and sentence dictation. The same averaging process was applied to z-scores, obtaining a general reading speed z-score, a general reading accuracy z-score and a general writing accuracy z-score.

A Bonferroni correction for multiple comparisons was applied in the correlation analysis between reading/writing and PUFA (three reading reading/writing general scores counting as one since they were mutually correlated at minimum r = 0.629, and seven independent PUFA related variables—total omega-3, total omega-6, and ratios omega-6/omega-3, AA/EPA, AA/DHA, AA/ALA, and LA/ALA—plus a total PUFA score expressing the sum of total omega-3 and total omega-6 (alpha/7 = 0.007)).

No Bonferroni correction was applied to the correlations between reading/writing variables and neuropsychological tests, since clear a priori hypotheses based on the literature predicted the associations between variables, and since such associations often concerned groups of mutually correlated variables.

For mediation models, which were constructed as post-hoc analyses based on the patterns of previously observed correlations, unidirectional hypotheses were considered, and one-tailed significance values were taken (alpha = 0.1).

## 3. Results

### 3.1. Participants’ Characteristics

Table 1 shows the general characteristics of the children with DD and the controls. A series of independent samples *t*-tests were computed to compare age, IQ, and reading and writing abilities (expressed as z-scores) in the two groups. As clearly shown, the two groups significantly differed in only the variables of interest, but all of them (see Table 1).

The distribution of reading and writing scores (expressed as z-scores) in the two groups is shown in Figure S2. From visual inspection of Figure S2a,b it can be easily observed that the two groups represent clearly distinct populations, but also that their performances can be rather homogeneously distributed along a continuum. Therefore, correlational analysis of the relationships with two additional groups of variables, expressing neuropsychological characteristics of the participants on the one hand and the blood levels of poly-unsaturated fatty acids on the other hand, should theoretically be possible without incurring the risk of spurious effects due to non-continuous distributions.

In order to perform such correlation analysis, the distributions of the other two groups of variables were also examined. Considering the blood levels of PUFA in the two groups of children, the distribution for TD children is characterized by lower variability as compared to DD children, with the exception of omega-6/omega-3 ratio, which is more similarly distributed (Figure S3). A series independent samples *t*-tests showed no differences between TD and DD groups in all measures of the blood levels of PUFA (all *p* > 0.075).

To the same end, the performances on the neuropsychological tests, which correlate significantly with PUFA and reading/writing, were plotted as separate histograms for the two groups so as to make comparison of the distributions easier. These graphs are reported in Figure S4. It can be observed that the data were not similarly distributed in the two groups. Moreover, cue effects in VSA tasks for accuracy were more variably distributed in the DD than in the TD group. Participants’ characteristics and performance profiles on reading/writing and neuropsychological tests are shown in Table 2. Data of all measures are expressed as raw scores.

### 3.2. Correlations between PUFA Levels and Reading and Writing Measures

The relationships between reading and writing scores (general reading time, general reading errors and general writing errors) and PUFA levels (n = 30) were analyzed by calculating partial correlations and controlling for age (Table 3). The AA/ALA ratio was positively correlated with DDE general reading time and with DDE general writing errors. The correlations with the component subtests of the two general scores were all significant (all *p* < 0.003). The LA/ALA ratio was positively correlated with DDE general reading time and with DDE general writing errors. No other PUFA-related values were associated with reading and writing performances. At a post-hoc analysis, ALA significantly correlated with both general reading time and general writing errors (r = −0.516, *p* = 0.004, and r = −0.394, *p* = 0.35, respectively) and the levels of AA and of ALA were not associated with any measure of interest (all *p* > 0.62).

### 3.3. Correlations between Reading and Writing Measures and Visual/Auditory Neuropsychological Tasks

As a second step, partial correlations controlling for age were computed between the reading and writing measures that had been found to correlate with PUFA ratios and the scores of the various neuropsychological tests in the two modalities, visual and auditory (Table 3). Phonological processing was found to be negatively associated with both DDE general reading time and DDE general writing errors. DDE general writing errors were also associated with the RAN measure and with left–right accuracy difference in VSA cue effect. The flanker effect time, expressed in seconds, was found to be positively correlated with DDE general reading time and DDE general writing errors. No significant associations emerged between reading and writing measures and any other measures of interest (r < 0.140, *p* > 0.468).

### 3.4. Correlations between PUFA Levels and Auditory/Visual Neuropsychological Functions

The last step of the correlation analyses was to assess the associations between auditory and visual neuropsychological functions that were found to correlate with reading and writing, and the PUFA ratios that were significantly correlated with reading and writing (Table 3).

Partial correlation analyses (with age as a control variable) highlighted a negative correlation of phonological processing with AA/ALA ratio, and with LA/ALA ratio, and positive correlations of the flanker effect with AA/ALA ratio, and LA/ALA ratio. The correlations of left–right accuracy difference in VSA cue effect with AA/ALA ratio and LA/ALA ratio were not statistically significant, although the last correlation (r = 0.316, *p* = 0.095) could be considered significant with respect to one-tailed significance (alpha = 0.1). Post-hoc analyses showed an association of AA with phonological processing (r = 0.392, *p* = 0.036) and of ALA with the flanker effect (r = −0.396, *p* = 0.033). RAN and the remaining auditory and visual neuropsychological measures did not show any significant correlations with PUFA (all *p* > 0.171).

### 3.5. Mediation Analyses

Based on previous correlation analyses, a series of analyses with GLM mediation models were conducted where reading speed and writing accuracy measures were used as dependent variables, the AA/ALA and LA/ALA ratios as independent variables, and the neuropsychological variables that were found to correlate with both the reading/writing scores and with the PUFA ratio as mediators. In the mediation models z-scores were used instead of raw scores, with the exception of PUFA ratios.

**Figure 1:**
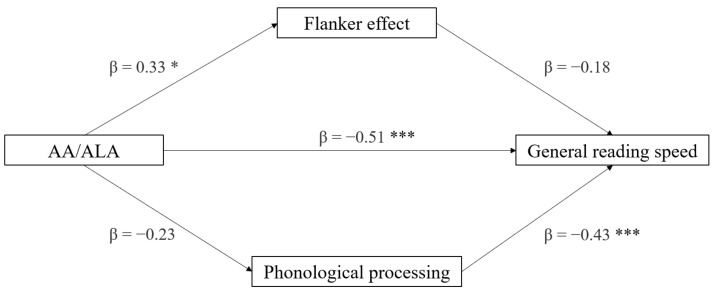
Mediation model with General reading speed (z-score) as dependent variable, AA/ALA ratio as independent variable, and neuropsychological variables as mediators (* *p* < 0.05, *** *p* < 0.001, one-tailed).

The first mediation model tested the roles of phonological processing and the flanker effect (mediators) in the association between AA/ALA ratio (independent variable) and DDE general reading speed (dependent variable) (see Figure 1). The indirect effect of AA/ALA on reading speed, through the phonological and neuropsychological measures, was not significant (*p* > 0.110). Only the direct effect was significant (*p* < 0.001), indicating an association between AA/ALA and reading ability independent of the neuropsychological mechanisms under consideration. In the component analysis, phonological processing was confirmed to be a significant predictor of reading speed (*p* < 0.001), and AA/ALA was found to predict flanker effect measures (*p* = 0.027). Considering the full regression model, the best predictor of reading speed was AA/ALA ratio (*p* < 0.001), followed by phonological processing (*p* = 0.001), and, not significantly, by the flanker effect (*p* = 0.093). Overall, the regression model was significant (*t* = −4.60, *p* < 0.001) (Table 4).

**Figure 2:**
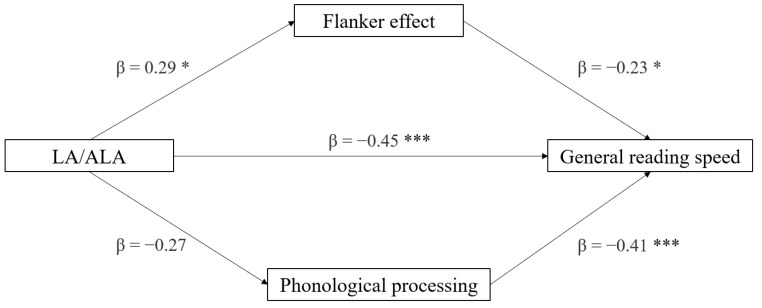
Mediation model with general reading speed (z-score) as the dependent variable, LA/ALA ratio as the independent variable, and neuropsychological variables as mediators (* *p* < 0.05, *** *p* < 0.001, one-tailed).

The second mediation model tested the roles of the same mediators in the effect of the LA/ALA ratio on reading speed (see Figure 2). Additionally, in this case only a direct effect of LA/ALA on reading ability emerged (*p* < 0.001), whereas the indirect effects were non-significant (*p* > 0.084). The most significant component predicting reading speed was phonological processing (*p* < 0.001), followed by the flanker effect (*p* = 0.033). In addition, the effect of LA/ALA on the flanker effect was significant (*p* = 0.047). Considering the full regression model, the best predictor of reading speed was LA:ALA (*p* = 0.001), followed by phonological processing (*p* = 0.003), and then (not significantly) the flanker effect (*p* = 0.058). Overall, the regression model was significant (*t* = −4.03, *p* < 0.001) (Table 4).

**Figure 3:**
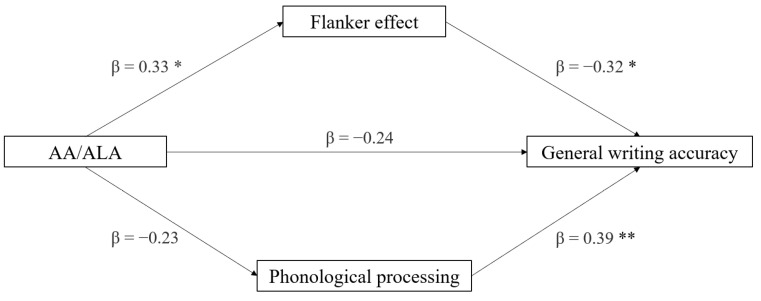
Mediation model with general writing accuracy (z-score) as the dependent variable, AA/ALA ratio as the independent variable, and neuropsychological variables as mediators (* *p* < 0.05, ** *p* < 0.01, one-tailed).

The third mediation model had DDE general writing accuracy as the dependent variable, the AA/ALA ratio as the independent variable, and the phonological processing and the flanker effect as mediators (see Figure 3). The indirect effects of AA and ALA on writing accuracy, through the neuropsychological measures, were not significant (*p* > 0.075). Additionally, the direct effect of AA:ALA on writing accuracy was not significant (*p* = 0.194). In the component analysis, phonological processing was confirmed to be a significant predictor of writing accuracy (*p* = 0.003) and the flanker effect (*p* = 0.016). AA:ALA was found to marginally predict the flanker effect (*p* = 0.027). In the full regression model, the best predictor of writing accuracy was phonological processing (*p* = 0.011), followed by the flanker effect (*p* = 0.035), and not significantly, by the AA/ALA ratio (*p* = 0.076). The regression model was significant (*t* = −2.42, *p* = 0.022) (Table 4).

**Figure 4:**
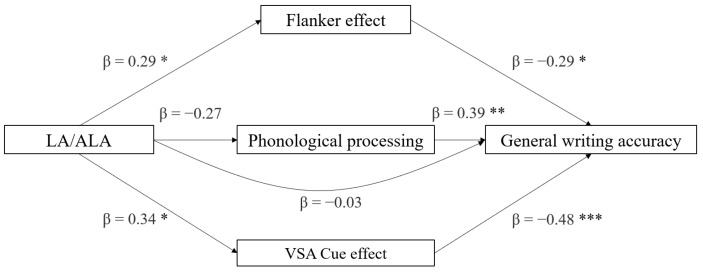
Mediation models with general writing accuracy (z-score) as the dependent variable, LA/ALA ratio as the independent variable, and neuropsychological variables as mediators (* *p* < 0.05, ** *p* < 0.01, *** *p* < 0.001, one-tailed).

In the fourth and last mediation model with writing accuracy, the effects of the LA/ALA ratio on writing accuracy were examined (see Figure 4). Among the indirect effects of LA/ALA on writing accuracy, the effect mediated by left–right accuracy difference in the cue effect was significant (*p* = 0.040) (Table 5) and the effects of the other two mediators were not found to be significant (*p* > 0.087). No direct effect of LA/ALA on writing accuracy emerged (*p* = 0.402). Considering the component effects, the most significant component predicting writing accuracy was the left–right accuracy difference in the cue effect (*p* < 0.001), followed by phonological processing (*p* = 0.001) and the flanker effect (*p* = 0.010). Moreover, LA/ALA was found to predict the cue effect (*p* = 0.024) and marginally the flanker effect (*p* = 0.047). In the full regression model, the best predictor of writing accuracy was the left–right accuracy difference in VSA cue effect (*p* = 0.001), followed by phonological processing (*p* = 0.006), and the flanker effect (*p* = 0.029). LA:ALA was confirmed to have no predictive power (*p* = 0.409). The regression model was significant (*t* = −2.07, *p* = 0.048) (Table 4).

## 4. Discussion

A series of analyses were conducted to explore the association of PUFA blood levels with reading and writing abilities, and to examine the influences of auditory and visual neuropsychological higher and lower-level functions on the associations between PUFA and learning abilities.

The correlation analysis showed significant associations of two omega-6 to omega-3 ratios with reading and writing measures, which are in line with expectations. Specifically, it was found that higher AA/ALA and LA/ALA ratios, but not AA/EPA or AA/DHA ratios, are associated with worse performances in reading speed and in writing accuracy. Significant associations were also found between reading and writing measures and some of the examined auditory and visual neuropsychological functions. High scores on phonological processing tests were associated with better performance in reading speed and writing accuracy, as predicted and as shown in many other research studies—e.g., [47,49].

Considering inhibition functions in VSA, correlation analyses showed an association of better performance in reading and writing tasks with a reduced visual flanker effect. In the same direction, better performance in the writing task was associated with higher speed in rapid automatized naming. The processes involved in rapid automatized naming and in phonological processing appear different. RAN tasks, for instance, require speeded retrieval of phonological codes from long-term memory and involve visual search processes and automatization of name retrieval [51,65,66]. Both phonological processing and RAN, however, are described as playing a central role in reading and writing [47,48,49,51].

Mediation models analyzing the direct and indirect effects of PUFA on reading and writing scores showed that the effects of fatty acids on learning measures appear to be direct rather than mediated by the visual and auditory neuropsychological mechanisms under investigation. The only significant indirect effect was found for the difference in accuracy between the left and right visual fields in visual-spatial cueing tasks, acting as a mediator for the effect of LA/ALA on writing accuracy. Regression analyses, by contrast, confirmed the roles of phonological awareness and other visual attentional factors as predictors of reading and writing skills. The above-mentioned effect confirms the role of VSA mechanisms in reading and writing abilities and suggests that visual low-level mechanisms may be more sensitive to the effects of favorable conditions related to the presence of higher omega-3 blood levels. More specifically, the asymmetry described for cueing tasks between the left and the right visual field is generally interpreted as an effect of altered attentional focusing [11,12], which would be reduced in the right visual field, producing very little effects of cueing, whereas it would be increased (or close-to normal) in the left visual field, an effect described by some authors as a “left minineglect” characterizing dyslexia [67,68]. While the flanker effect also measures attentional mechanisms related to focusing and inhibition and to the ability to control crowding effects, the specific information conveyed by the asymmetry of focusing in the cueing task seems to be particularly relevant to reading and dyslexia. Indeed, asymmetries in the distribution of visual-perceptual (or visual-attentional) processing have also been described following different theories and paradigms which put such asymmetry in relation with directional aspects in the learning-to-read process [69,70]. A recent study [71] showed multiple and independent pieces of evidence about a causal link between crowding and reading acquisition, suggesting the role of VSA in crowding. This type of attentional mechanism was clearly shown to be involved in both reading and writing processes in the present study; however, it turned out to be a (nearly significant) mediator of the effect of PUFA levels for writing accuracy only.

On the whole, then, the relationships of two specific ratios of omega-6/omega-3 (AA/ALA and LA/ALA) with reading and writing skills emerged very clearly in the results of our study.

Since none of the neuropsychological mechanisms which are known to affect reading and writing seems to be mediating such a relationship, a series of possible explanations can be hypothesized. First of all, these findings could be explained by a non-specific effect of PUFA on reading and writing abilities, linked to the anti-inflammatory function that is usually described for omega-3 fatty acids. Surprisingly, the ratio AA/ALA but not the AA/EPA or AA/DHA ratios, showed correlations with reading and writing performances. The explanation for an impact by the parent omega-3 rather than the longer derivatives is not immediately clear, and similar results were not observed in the literature. One report in vitro [72] showed that ALA has neuroprotective effects, and another ongoing trial on the effect of walnuts, rich in ALA among other bioactive substances, is trying to better understand the role of plant-based omega-3 PUFA in neuropsychological development during adolescence [73]. Research has shown, moreover, that the omega-6 PUFA LA and AA are important in cognitive function but are pro-inflammatory [29]. A high omega-6 to omega-3 ratio (in favor of higher levels of omega-6), then, could be associated with a reduction in learning efficiency and thus with worse performance in reading and writing skills. The present results are in line with those of a previous study by Cyhlarova and colleagues [25], showing positive correlations between total omega-3 concentrations and reading performance in both adults with dyslexia and control participants, a negative correlation (trend) between total omega-6 and reading in a dyslexic group, and negative correlations between both omega-6/omega-3 and LA/ALA ratios and reading. A further series of correlations were computed to better understand the reasons for significant effects emerging for the less frequently reported ratios AA/ALA and LA/ALA and not for more commonly reported indices, such as omega-6/omega-3, the total level of omega-3, and total PUFA. It was observed that, indeed, none of the two ratios AA/ALA and LA/ALA was at all related to the above mentioned frequently reported ratios (all r ≤ 0.164, all *p* ≤ 0.395), confirming that the ratios emerging in the present study represent completely different aspects of the PUFA profile. Even if the first ratio involved AA, representing a large proportion of omega-6 acids, a post-hoc check revealed that partial correlations of reading/writing general scores with AA did not reach significance (r ≤ 0.351, *p* ≥ 0.062), confirming that the ratio is more relevant than absolute PUFA levels in the blood. LA/ALA, on the other hand, was not at all correlated with AA, nor with any other fatty acid considered in the present study, again suggesting possible specific effects (to be further investigated). It should be noted, moreover, that the correlations in the present study suggest that the effects of AA/ALA and LA/ALA on reading and writing abilities are mainly mediated by ALA (r = −0.394 and −0.516 with writing errors and reading time, respectively, *p* < 0.035), and to a lesser extent by AA (for which the only correlation approaching significance is with reading time, r = 0.351, *p* = 0.062) and LA (no significant correlations, all r ≤ 0.120).

An ad hoc search of the literature highlighted that, even if no specific effects of AA/ALA and LA/ALA have been reported till now for reading skills and DD, inverse associations were observed between maternal intake of LA/ALA and mental and psychomotor development in 6-month-old infants [74]. Another study reported that a high maternal breast milk LA percent (>9.7% of fatty acids) was associated with reduced motor and cognitive scores in 2–3-year-old infants [75] and with reduced verbal IQ at 5 to 6 years of age [76]; and Lassek and Gaulin [77] found an inverse correlation between breast milk LA percent composition and cognitive scores in 15-year-old children, suggesting a long-lasting impact of maternal LA on offspring cognitive skills. Moreover, high LA/ALA ratios seem to modulate immune responses through T-cell proliferation [78] in adult men, whereas lower levels of AA but higher levels of ALA were found to be associated with better performance in verbal learning and memory tasks in post-ischemic patients. Finally, ALA supplementation was shown to improve hippocampal neurons survival, memory, and spatial learning after ischemic stroke in an animal study [79]. Interestingly, despite being a major part of the diet, LA is mainly viewed as an essential precursor to AA, and little is known about the mechanisms through which it affects the brain. This is also due to its low concentration (<2% of total fatty acids) compared to DHA and AA [80]. Nonetheless, recent studies suggested that the effects of LA in the brain could be mediated by “oxidized linoleic acid metabolites” (OXLAMs), lipid mediators known to regulate pain and inflammatory signaling in peripheral tissue [81]. Based on this evidence, we decided to perform additional analyses exploring the possibility that working memory (for which index scores were available from the WISC-IV only for the children with DD) could act as a mediator between PUFA ratios and reading/writing performance. Indeed, high correlations were present both between the ratios and WM, and between WM scores and reading and writing scores. Nonetheless, mediation analyses once more revealed only direct effects of the PUFA on reading and writing, with no significant indirect effects mediated by WM.

A last check included the two ratios AA/ALA and LA/ALA and cognitive abilities. While no significant associations were found with full-scale IQ, interesting results were obtained considering the subtests of the WISC. In particular, significant associations emerged between the two PUFA ratios and the working memory index (r = −0.708, *p* = 0.007 with AA/ALA, r = −0.597, *p* = 0.031 with LA/ALA ratio). Post-hoc analyses revealed significant correlations with AA and ALA (*p* < 0.032). However, working memory measures were available for only a part of the participants (with DD), and further studies should allow better characterization of such associations with larger samples. Indeed, several studies investigated the association between working memory and dietary intake, specifically omega-3 and omega-6, but results were variable [82].

A second possible reason for the absence of mediation by the hypothesized neuropsychological mechanisms is that the effects hypothesized to play a role in reading and writing skills differ in children with typical development with respect to children with DD, and may mask each other when both groups are examined together. Indeed, the decision to consider both groups as a whole depended on the observation that the reading and writing skills were rather evenly distributed in our sample, i.e., they did not constitute two clearly separate groups but rather aligned along a continuum from good to impaired abilities. Nonetheless, as stated above, the mechanisms causing such (dis)abilities might still differ in the two groups. If such a hypothesis were true, it would be necessary to run separate analyses in the two subgroups, but the size of the present sample did not allow for this operation. The small size of the sample can thus be considered as a limitation of the study. Further limitations include the cross-sectional nature of the present study (at least at this stage), not allowing us to draw any clear conclusions about the causal direction of observed relationships. Even if a hypothetical causal chain is not equally probable in the two directions (while it is reasonable to hypothesize that PUFA levels in the blood have some influence on reading and writing abilities, it is less likely that reading and writing abilities have an influence on fatty acids), causation is just a hypothesis. The use of mediation models, evaluating the statistical tenability of a directional relationship from the independent variable to the dependent variable, through the action of mediators, may add strength to the hypothesis (in the present case, disconfirming rather confirming the role of hypothesized mediators), but cannot prove any direct causal role.

Furthermore, it should be noted that, as stated in the section about correlation analysis, we did not find the expected significant association of learning abilities and PUFA levels with some of the neuropsychological tests, such as visual search, motion perception, and auditory rhythm discrimination. Overall, we had expected much stronger and more significant associations, as reported in other published studies [11,19,20,57].

Potential explanations for these differences could be, once more, related to the small sample size and the inclusion of both TD and DD children in the same analysis. Even if it is largely agreed that the two groups constitute a continuum [83] rather than clearly separate groups (also considering that cut-offs for diagnosis are rather arbitrary and largely vary from study to study), as also discussed by Cilibrasi and Tsimpli [84], the effects of impairments in visual and auditory neuropsychological functions since the first stages of development might determine increasingly different developmental trajectories though changes in the organization of cognitive and neurobiological systems. Such changes might alter the mutual relationships between functions (or their strengths), and compensatory processes might call into play additional mechanisms [85,86], which could result in making the two groups even more different. Such a broad and complex range of possibilities should be always kept in mind when observing and interpreting differences in populations of children with neurodevelopmental disorders, and great caution should be used in drawing any conclusion about the stability and generalizability of such differences. Age and sex-related differences, among others, should be carefully evaluated and interpreted in the framework of growth mechanisms and of changing environmental and biological (e.g., hormonal) conditions. Additionally, immunological factors are suggested (considering the crucial role of specific fatty acids such as AA, LA, and ALA) to be possibly involved in determining developmental outcomes, always dynamically. Disentangling all these factors and mechanisms is clearly not a trivial enterprise, and requires time and very rigorous designs with thorough investigations. Furthermore, language (or orthography) specific factors such as orthographic transparency could play a role in the present and future results and should be taken into account. For instance, the crucial role shown to be played by phonological awareness (phonological processing) but not by RAN in the present results could be due to the shallow nature of Italian orthography, as could also the role of visual-attentional mechanisms. Both processes in fact could be involved in letter-by-letter or syllable-by-syllable scanning/assembling in a sublexical reading approach, made possible by the high regularity of Italian.

Following this exploratory investigation, the larger project of which the present study constitutes the first step, and other future studies on larger samples may be able to provide clearer answers to some of these unsolved questions.

## 5. Conclusions

Two important findings emerged from the present study. The first one was the confirmation of a general, direct relationship between PUFA and reading/writing skills, regardless of the presence of specific reading difficulties. The second one was the absence of the hypothesized mediating role of neuropsychological functions as a link between PUFA levels or PUFA ratios and reading and writing performance. Even if specific pathways, possibly differing between TD and DD children, may be identified in larger samples, the results in the overall sample do not show clear effects of PUFA on neuropsychological functions that are then expressed in literacy acquisition, and rather point to other effects of PUFA on cognitive functions, to be clarified in future studies and analyses.

## Figures and Tables

**Table 1:** Descriptive statistics and results of independent samples *t*-test comparisons of age, IQ, and reading and writing scores for children with (DD) vs. without (TD) a diagnosis of developmental dyslexia.

	DD Children n = 15	TD Children n = 15	Group Comparison
	Mean (SD)	Mean (SD)	Independent Samples *t*-Test: *t*, *p*
Age	10.96 (1.33)	10.70 (1.56)	0.494, 0.625
IQ	103.47 (9.49)	104.20 (14.99)	−0.160, 0.874
General reading accuracy	−1.80 (1.13)	0.56 (0.44)	−7.567, <0.001
General reading speed	−3.71 (2.71)	0.10 (0.65)	−5.287, <0.001
General writing accuracy	−2.20 (2.49)	0.43 (0.50)	−4.004, <0.001

**Table 2:** Participants’ characteristics and performance profiles in reading/writing and neuropsychological measures of interest. Results are expressed as raw scores.

	Mean (SD)	Range
Age	10.83 (1.43)	8.17–13.58
IQ	103.83 (12.33)	85–125
General reading errors	5.83 (5.28)	0–21.50
General reading time (s)	127.46 (89.07)	55–498.50
General writing errors	3.03 (3.21)	0–12.50
Rhythmic pattern discrimination (Pattern total accuracy)	19.03 (4.98)	8–24
Phonological processing accuracy	46.70 (4.22)	35–52
rapid automatized naming errors	0.83 (1.51)	0–6
Rapid automatized naming time (s)	84.75 (18.24)	57.87–138
Flanker effect time (s)	85.93 (93.69)	−189–288
VSA cue effect accuracy	0.50 (1.65)	−3–4.50
VSA cue effect speed (s)	−3.51 (79.42)	−130.63–294.25
Motion coherence accuracy	31.07 (4.73)	23–38
Visual search errors	0.30 (0.60)	0–2
Visual search time (s)	23.01 (7.51)	13.10–39.68

**Table 3:** Pearson’s partial correlations (controlling for Age; n = 30, df = 27) between PUFA ratios, reading/writing measures and auditory/visual neuropsychological functions (* *p* < 0.05, ** *p* < 0.01, *** *p* < 0.001).

	1	2	3	4	5	6	7	8
1. AA/ALA ratio	-							
2. LA/ALA ratio	0.949 ***	-						
3. General reading time (s)	0.807 ***	0.763 ***	-					
4. General writing errors	0.550 **	0.522 **	0.794 ***	-				
5. Phonological processing accuracy	−0.416 *	−0.440 *	−0.670 ***	−0.658 ***	-			
6. Rapid automatized naming time (s)	0.117	0.065	0.277	0.396 *	−0.246	-		
7. Flanker effect time (s)	0.540 **	0.470 *	0.569 **	0.644 ***	−0.351	0.224	-	
8. VSA cue effect accuracy	0.287	0.316	0.242	0.426 *	0.426 *	0.074	0.291	-

**Table 4:** Regression analysis predicting general reading speed and general writing accuracy.

Effect	Variable	Estimate	SE	Lower	Upper	β	df	*t*	*p*
AA/ALA	General reading speed	−0.044	0.010	−0.063	−0.024	−0.656	28	−4.60	<0.001
LA/ALA		−0.021	0.005	−0.032	−0.010	−0.606	28	−4.03	<0.001
AA/ALA	General writing accuracy	−0.023	0.010	−0.042	−0.003	−0.416	28	−2.42	0.022
LA/ALA		−0.010	0.005	−0.020	−1.11 × 10^−4^	−0.364	28	−2.07	0.048

**Table 5:** Significant indirect effect of the mediation model for general writing accuracy (one-tailed).

Effect	Estimate	SE	Lower	Upper	β	z	*p*
LA/ALA ⇒ VSA cue effect ⇒ General writing accuracy	−0.004	0.002	−0.010	5.14 × 10^−4^	−0.163	−1.750	0.080

## Data Availability

Data are not available due to the ongoing main project n. 677.

## Supplementary Materials

The following supporting information can be downloaded at: www.mdpi.com/article/10.3390/brainsci12020169/s1, Figure S1: Distributions of raw scores for general reading errors, general reading time, and general writing errors; Figure S2: Distributions of z-scores for general reading accuracy, general reading speed, and general writing accuracy; Figure S3: Distributions of the blood levels of total PUFA, total omega-6, total omega-3, omega-6/omega-3 ratio, AA/ALA ratio, and LA/ALA ratio; Figure S4: Distribution of the performances on the neuropsychological tests, phonological awareness, rapid automatized naming, flanker effect, cue effects in visual spatial attention.
