# Supplementary material for: Direct and Indirect Effects of Blood Levels of Omega-3 and Omega-6 Fatty Acids on Reading and Writing (Dis)Abilities
Source: Brain Sci. 2022 Jan 27;12(2):169. doi: 10.3390/brainsci12020169 (PMC8870518; doi:10.3390/brainsci12020169)
Supplement: Supplementary file 1 [file brainsci-12-00169-s001.zip › brainsci-1512517 -Supplementary materials.pdf]

brain sciences

MDPI

Supplementary materials

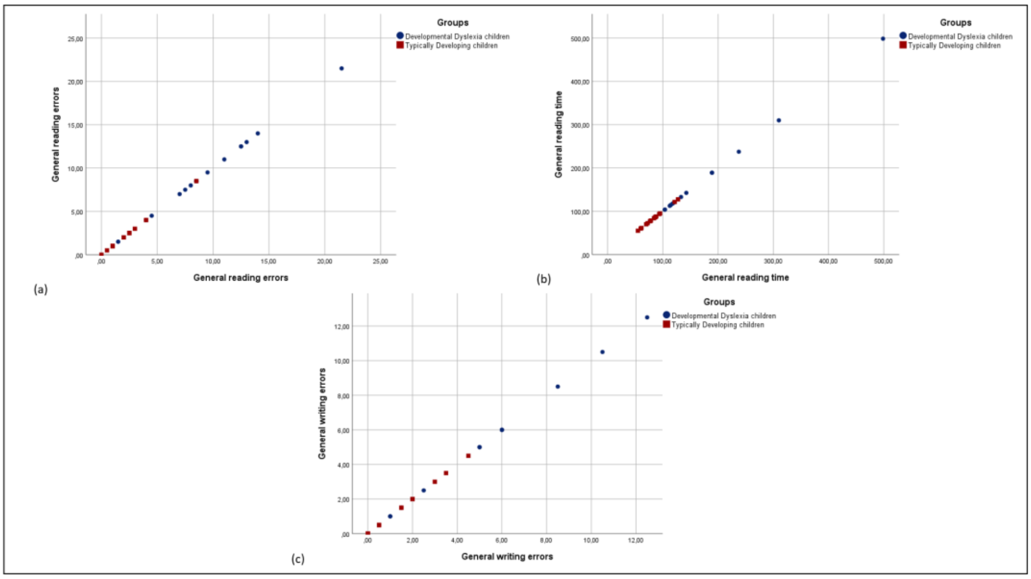

Figure S1. Distributions of raw scores for general reading errors (a), general reading time (b), and general writing errors (c). Blue dots represent children with developmental dyslexia (DD); red dots represent typically developing children (TD).

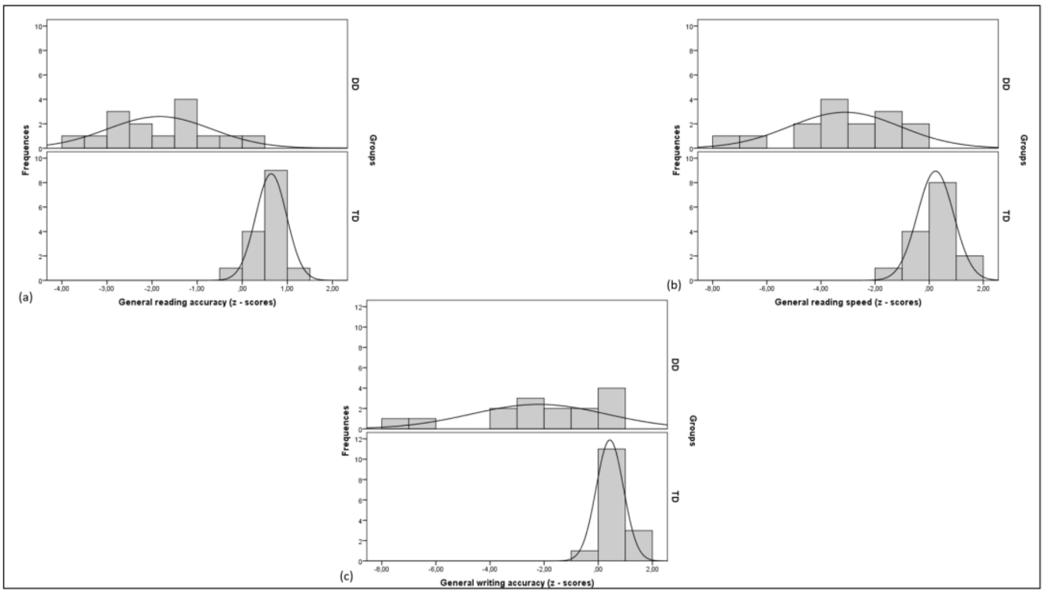

Figure S2. Distributions of z-scores for general reading accuracy (a), general reading speed (b), and general writing accuracy (c).

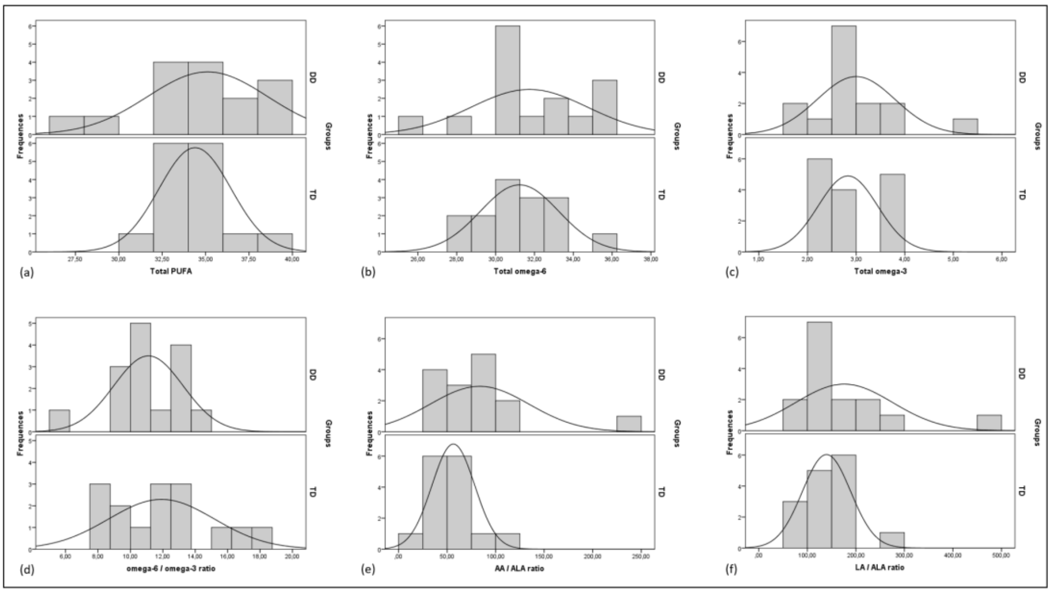

Figure S3. Distributions of the blood levels of total PUFA (a), total omega-6 (b), total omega-3 (c), omega-6/omega-3 ratio (d), AA/ALA ratio (e), and LA/ALA ratio (f).

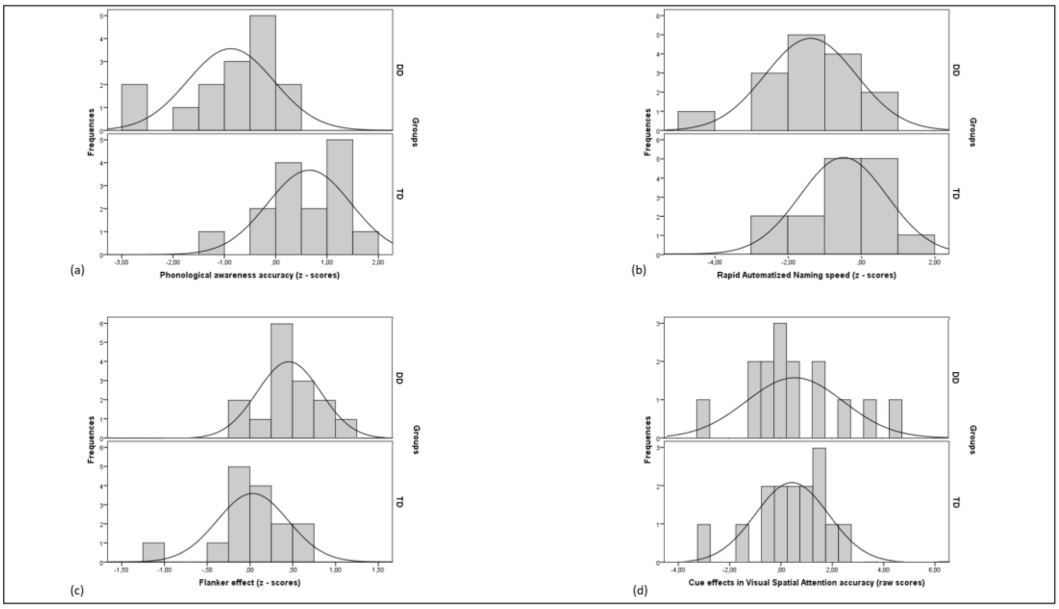

Figure S4. Distribution of the performances on the neuropsychological tests, phonological awareness (a), rapid automatized naming (b), flanker effect (c), cue effects in visual spatial attention (d).
